# The paradox of early pregnancy care: Overtreatment amid systemic neglect

**DOI:** 10.1111/aogs.70097

**Published:** 2025-11-17

**Authors:** Tina Tellum, Joel Naftalin

**Affiliations:** ^1^ Department of Gynecology Oslo University Hospital Oslo Norway; ^2^ Institute of Clinical Medicine, Faculty of Medicine University of Oslo Oslo Norway; ^3^ Early Pregnancy Unit University College London Hospitals NHS Foundation Trust London UK

Experiencing complications in early pregnancy is common. Miscarriage alone occurs in about 15%–20% of recognized pregnancies, amounting to 23 million annually worldwide^1^ and represents in a biological sense, a natural event. However, while medically intending to convey frequency, doctors inappropriately call it “normal,” potentially minimizing the significant psychological impact it can have for the affected patient and their partner.^2^ Incomplete miscarriages, with residual tissue causing bleeding, infection, or adhesions, often require repeated follow‐up and treatment. Ectopic pregnancy, affecting 1%–2% of pregnancies, is another major early pregnancy complication that places a heavy burden on patients and healthcare systems through high resource use and costs. Further, it must be acknowledged that while deaths from early pregnancy complications are thankfully rare, they do still occur, with a disproportionate number occurring in the developing world.^3^

Despite this considerable individual, societal, and economic burden, early pregnancy care remains underdeveloped, perhaps because it is perceived as straightforward, with the majority of care being provided by junior doctors. It stands at a crossroads, torn between the allure of advanced surgical techniques and the persistent reality of systemic underinvestment and insufficient training. This dissonance perpetuates uneven, sometimes substandard care, leaving patients vulnerable to both unnecessary interventions and basic oversights.

## 1 UNDER‐ AND MISDIAGNOSIS

An old saying among gynecologists goes: “You see the heartbeat before the embryo.” While often true, it takes considerable skill to exclude a heartbeat with certainty and sometimes it is simply not possible, as the gestation could be too early. Yet, an “empty” gestational sac or an embryo without cardiac activity can be wrongly interpreted as a definite miscarriage rather than as a limitation of scanning skills, creating so‐called “Lazarus” embryos: pregnancies initially declared lost but later found viable—with an unmeasured number tragically unintentionally terminated.^4^, ^5^ To avoid such errors, diagnostic criteria for miscarriage had to be set extremely wide, building in a safety margin that prioritized preventing the most catastrophic mistake: terminating a viable pregnancy.^4^ Worryingly, adherence to guidelines is variable.^6^

A marker of ultrasound quality at a department is the rate of pregnancies of unknown location (PUL). PUL is defined as a positive pregnancy test without a detectable pregnancy on ultrasound. While very early pregnancies may not yet be visible, an expert operator can usually localize a pregnancy when serum hCG (s‐hCG) levels exceed 1000 IU/L. A PUL rate of up to 20% is considered acceptable, with some expert centers reporting rates as low as 8%.^7^ Yet, some centers report rates as high as 42%, which is clear evidence of poor quality ultrasound even in units dedicated to early pregnancy care.^8^ Strikingly, most departments do not monitor their PUL rates, reflecting poor quality control.

Despite this, standardized ultrasound training for early pregnancy care is generally lacking, which is in sharp contrast to the extensive certification systems in fetal–maternal ultrasound that are widely established.

Misdiagnosis is further compounded by the misuse of biochemistry. s‐hCG has wide physiological variation and may even decline in normally developing pregnancies.^9^ A single or serial s‐hCG measurement should never be used to diagnose miscarriage or determine pregnancy location; yet many women still receive definitive judgments on this basis, again potentially leading them to unwittingly terminate an ongoing, wanted pregnancy. While s‐hCG has a role in *managing* ectopic pregnancy, its values overlap substantially with those of miscarriage and normal gestation and are, therefore, not diagnostically useful, a fact not universally recognized.^3^ In diagnosing retained products of conception (RPOC), hCG is largely unhelpful: levels can normalize despite the presence of clinically significant tissue, and levels may still be positive despite no tissue remaining.^10^ Nevertheless, s‐hCG testing continues to be used routinely in an inappropriate manner, reflecting a lack of knowledge of early pregnancy pathophysiology and misguided trust in biochemistry.

Paradoxically, while s‐hCG is overused, progesterone, an inexpensive marker that indicates how quickly a pregnancy will develop, can in combination with s‐hCG reliably distinguish viable or potentially harmful ectopic from failing pregnancies. Using the widely validated triage tools that are based on ultrasound, progesterone and s‐hCG could streamline PUL triage^11^ but are rarely used in routine care across the Nordic countries and much of the world. This results in repeated follow‐up visits, unnecessary anxiety for patients, and wasteful use of healthcare resources.

## 2 THE HYSTEROSCOPY DEBATE: PRECISION VERSUS OVERKILL

Hysteroscopic management of retained products of conception (RPOC) has gained popularity as a safer alternative to “blind” curettage, with claims of lower adhesion rates and fewer cases of residual tissue, and is often promoted as the superior method of choice.^12^ The evidence however does not substantiate these claims. Two frequently cited studies found no increased risk of adhesions or operative complications with either method.^13^, ^14^ One study did show more residual tissue after vacuum aspiration, but the trial was heavily imbalanced in group size (124 patients undergoing hysteroscopy vs 28 undergoing vacuum aspiration), raising the question of whether operator skill may have influenced the results.^13^

At the same time, important limitations of hysteroscopy are often overlooked. In the cited trials,^13^, ^14^ only RPOC sizes up to 4 cm were included, which is likely the upper technical limit of hysteroscopy. By contrast, ultrasound‐guided vacuum curettage or forceps evacuation has no such limit. Another RCT reported that 7% of hysteroscopic procedures could not be completed compared to 0% in the vacuum aspiration group, with no difference in subsequent pregnancy outcomes or time to conception.^15^

It is misleading to call ultrasound‐guided vacuum curettage “blind.” Under ultrasound guidance, the position of the instrument is visible at all times, and when performed correctly, the risk of perforation is negligible. In hysteroscopy, however, adhesions, bleeding, or abnormalities can obscure safe visualization, myometrial thickness behind the RPOC cannot be assessed, and RPOC located laterally, where the uterine wall is thinnest and the arcuate vessels are near, pose a real risk of perforation and other serious complications.^11^, ^12^ Visibility is especially reduced in vascularized RPOC due to bleeding, often requiring procedures to be postponed for 6–8 weeks until vascularity decreases. In the presence of infection, hysteroscopy is relatively contraindicated due to the significant risks of spreading infection either hematogenous or directly to the pelvic through the Fallopian tubes. In contrast, ultrasound‐guided removal does not have this risk and can be performed immediately, without a wait.

The psychological burden of protracted miscarriage management should not be underestimated. Most women prefer timely and effective treatment to shorten recovery and psychological distress and attempt a new pregnancy without delay. The long waiting times required for safer hysteroscopic management are rarely addressed in studies, nor are patients' perspectives included.^13^, ^14^, ^15^ Instead, one‐armed studies continue to focus exclusively on various hysteroscopic instruments while disregarding ultrasound‐guided evacuation, creating a self‐reinforcing narrative.^12^

Ultrasound‐guided evacuation remains underutilized despite clear advantages: no need for specialized equipment, lower costs (especially with manual vacuum aspiration), shorter operating times, suitability for non‐tertiary and low‐resource settings, and immediate availability. Continuing to ignore these benefits risks promoting a “one‐size‐fits‐all” approach that favors complexity over patient‐centered care and leads to the loss of important skills in doctors.

## 3 CESAREAN SCAR PREGNANCIES: THE ALLURE OF SURGICAL COMPLEXITY

Cesarean scar pregnancy (CSP) management illustrates the same pattern. When diagnosed early, CSP can be treated safely and effectively in the first trimester, but if allowed to progress, it may result in severe maternal morbidity through the development of placenta accreta spectrum. Laparoscopic excision and hysteroscopic resection are increasingly promoted as “probably the most effective” methods based on small studies reporting high success rates,^16^ despite contradictory evidence.^3^, ^17^, ^18^

Laparoscopy and hysteroscopy for CSP management demand advanced surgical skills and carry serious risks including hemorrhage and bladder injury.^18^ In contrast, ultrasound‐guided suction evacuation offers high success rates, low complication rates, and substantially lower costs,^17^, ^18^ but is frequently overlooked. While laparoscopy may be the best option in rare CSP cases not accessible transcervically, the frequent escalation to endoscopic surgery, sometimes combined with both methotrexate and hysteroscopy,^19^ even for early and uncomplicated cases, reflects a broader culture of overcomplicating surgical management when simpler and safer options are available. At the same time, limited understanding of CSP risk stratification, factors such as myometrial thickness, vascularization and location, together with delayed diagnosis contribute to inappropriate management, ranging from excessive intervention to ineffective expectant care.^3^, ^17^

## 4 SYSTEMIC NEGLECT: THE SILENT CRISIS

Beneath this obsession with novelty, possibly egged on by companies with vested interests, lies a fractured system. Early pregnancy complications are frequently managed by junior doctors lacking specialized training. Misdiagnosis and mismanagement of ectopic pregnancies and miscarriages often stem from inadequate ultrasound skills or knowledge gaps in pathophysiology.^20^

A viable infrastructure for early pregnancy care is absent: most countries including the Nordic countries lack early pregnancy assessment units (EPAU). The United Kingdom is no utopia for healthcare, but it does have a network of over 200 early pregnancy units providing specialized compassionate care. Streamlined care and access to EPAU that experience vaginal bleeding or pain in early pregnancy alleviate psychological stress and avoid substandard and disrespectful care.^21^, ^22^ Specialized EPAU have also been shown to reduce both costs and inpatient admissions.^23^, ^24^

Working toward balanced, equitable care requires addressing these disparities:

1. *High‐quality ultrasound as standard*: Acknowledge that high‐quality ultrasound evaluation is the bedrock of high‐quality early pregnancy care and establish systematic, universal training.
2. *Invest in foundations*: Mandate early pregnancy ultrasound accreditation, establish EPAUs, and implement evidence‐based standardized protocols to reduce diagnostic errors.
3. *De‐escalate when possible*: Reserve advanced techniques for selected cases while prioritizing ultrasound‐guided methods for straightforward RPOC and CSP.
4. *Center patient voices*: As highlighted in EPAU evaluations, compassionate communication and continuity of care are as critical as surgical precision.

The field must reconcile its fascination with innovation with the urgent need for equitable, evidence‐based care. Until then, early pregnancy management will remain a tale of two extremes: overcomplicated surgeries for some, and neglect for many.

## AUTHOR CONTRIBUTIONS

Tina Tellum conceptualized the editorial, conducted the literature review, and wrote the first draft. Joel Naftalin provided critical review. All authors approved the final version for publication.

## CONFLICT OF INTEREST STATEMENT

JN is a member of the executive committee of the Association of Early Pregnancy Units (United Kingdom). TT declares direct or indirect speaker fees for lectures on gynecological ultrasound from GE, Samsung, Gedeon Richter and Olympus.

## Data Availability

Data sharing not applicable to this article as no datasets were generated or analyzed.
